# Morphologies and Structure of Brain Lipid Membrane Dispersions

**DOI:** 10.3389/fcell.2021.675140

**Published:** 2021-06-14

**Authors:** Viveka Alfredsson, Pierandrea Lo Nostro, Barry Ninham, Tommy Nylander

**Affiliations:** ^1^Centre for Analysis and Synthesis, Department of Chemistry, Lund University, Lund, Sweden; ^2^Department of Chemistry “Ugo Schiff” and CSGI, University of Florence, Sesto Fiorentino, Italy; ^3^Department of Applied Mathematics, Research School of Physical Sciences and Engineering, Australian National University, Canberra, ACT, Australia; ^4^Physical Chemistry, Department of Chemistry, Lund University, Lund, Sweden; ^5^NanoLund, Lund University, Lund, Sweden; ^6^Lund Institute of Advanced Neutron and X-ray Science, Lund, Sweden

**Keywords:** brain lipid, sphingomyelin, cholesterol, structure and morphology, specific ion effects, X-ray diffraction, cryo-TEM

## Abstract

This study aims to explore the variety of previously unknown morphologies that brain lipids form in aqueous solutions. We study how these structures are dependent on cholesterol content, salt solution composition, and temperature. For this purpose, dispersions of porcine sphingomyelin with varying amounts of cholesterol as well as dispersions of porcine brain lipid extracts were investigated. We used cryo-TEM to investigate the dispersions at high-salt solution content together with small-angle (SAXD) and wide-angle X-ray diffraction (WAXD) and differential scanning calorimetry (DSC) for dispersions in the corresponding salt solution at high lipid content. Sphingomyelin forms multilamellar vesicles in large excess of aqueous salt solution. These vesicles appear as double rippled bilayers in the images and as split Bragg peaks in SAXD together with a very distinct lamellar phase pattern. These features disappear with increasing temperature, and addition of cholesterol as the WAXD data shows that the peak corresponding to the chain crystallinity disappears. The dispersions of sphingomyelin at high cholesterol content form large vesicular type of structures with smooth bilayers. The repeat distance of the lamellar phase depends on temperature, salt solution composition, and slightly with cholesterol content. The brain lipid extracts form large multilamellar vesicles often attached to assemblies of higher electron density. We think that this is probably an example of supra self-assembly with a multiple-layered vesicle surrounding an interior cubic microphase. This is challenging to resolve. DSC shows the presence of different kinds of water bound to the lipid aggregates as a function of the lipid content. Comparison with the effect of lithium, sodium, and calcium salts on the structural parameters of the sphingomyelin and the morphologies of brain lipid extract morphologies demonstrate that lithium has remarkable effects also at low content.

## Introduction

The structure—function relationship of brain tissue is poorly understood. The brain structure is a complex design based on a rich variety of lipids, assembled into intriguing structures, crucial to their function in, e.g., signal transduction that sometimes involved coupled proteins (Agranoff et al., 1999). Theories of self-assembly involving lipids (Israelachvili et al., 1976; Larsson, 1989; Hyde et al., 1997; Ninham et al., 2017a,b) depend mainly on two parameters. One is local curvature at the lipid—aqueous interface set by the balance of head group vs. hydrocarbon tail forces. This is pivotal in many biological systems (Israelachvili et al., 1976; Hyde et al., 1997). It can be characterized by the packing parameter introduced by the Ninham group in the 1970s (Israelachvili et al., 1976; Mitchell and Ninham, 1981). The packing parameter (v/al) considers the ratio between the volume of the hydrophobic chain (v) and the product of the cross-section head group area (a) and the chain length in its fully stretched conformation (l). This concept, originally mostly used to describe “simpler” surfactant and lipid self-assembly system structures (Ninham et al., 2017a), has recently also been found to be useful to describe more complex biological systems (Lauwers et al., 2016; Ninham et al., 2017b). The other crucial determinant of structure is that of global packing constraints. These include geometric and molecular forces between aggregates (Israelachvili et al., 1976; Hyde et al., 1997).

The brain lipid system is quite diverse, both in the headgroup and acyl chain distribution. These are partly polyunsaturated fatty acid (PUFA) chains (Lauwers et al., 2016). The role of lipids with polyunsaturated chains in providing curvature that can facilitate presynaptic function has been pointed out (Antonny et al., 2015). This effect can also be assigned to their higher conformational flexibility. Here, it is interesting to point out that a particular type of phospholipids, plasmenyl-phospholipids (plasmalogens), which are ether-type glycerophospholipids with polyunsaturated chains are present in brain lipids. They have been reported to induce intriguing supramolecular structures (Angelova et al., 2021). Brain lipid extracts usually contains a large fraction of cholesterol, reported to be nearly 23% in the gray matter (Pasquini and Soto, 1972; Dreissig et al., 2009). In this context, it is important to emphasize that cholesterol is known to affect membrane fluidity and packing. Earlier studies show that the main effect of adding cholesterol is on the acyl chain region close to the polar headgroup, leading to higher rigidity and a thicker bilayer for dilaurylphosphatidylcholine (DLPC) and dipalmitoylphosphatidylcholine (DMPC) (McIntosh, 1978). It should here be noted that for polyunsaturated phosphatidylcholine, cholesterol was found to lie flat in the middle of the bilayer (Harroun et al., 2008). The consequence of increasing the stiffness of a curved lipid bilayer is to decrease the packing parameter and, hence, the negative curvature of the lipid bilayer. This has been shown to lead to swelling of a bicontinuous cubic phase in excess water (Tyler et al., 2015). Similar results were obtained in the study of the adsorption of a strong hydrophobe in the lamellar phase of dioleoylphosphatidylcholine (DOPC) (Borsacchi et al., 2016). Cholesterol has been shown to lower the phase transition temperature from gel to liquid crystalline lamellar phase (L_α_) of dimyristoylphosphatidylcholine (DMPC) from about 22°C to about 5°C (Mortensen et al., 1988). Interestingly, it was found that at lower content of cholesterol, typically below 14%, rippled bilayer gel phase structure, P_β_, is stabilized at temperatures below 22°C. Hjort Ipsen et al. (1987) have investigated in detail the DPPC-cholesterol aqueous phase behavior using theoretical modeling to provide deeper insight. One of the characteristics of this system is the fact that it features a two-phase regime below between about 10 and 20 mol% cholesterol. Below 40°C, this is a solid ordered and liquid ordered phase, while above this temperature, this is a coexisting liquid disordered and a liquid ordered phase. At lower cholesterol content, this is a single-phase regime, with a solid (gel) phase below 40°C and a liquid disordered phase at higher temperatures. For more than 20 mol%, the liquid ordered phase prevails. It is clear that cholesterol disturbs the order of the gel phase. The existence of phase separation in biomimetic lipid mixtures has triggered debates since the 1970s on whether 2D phase separated domains of so-called “lipid rafts” exist and whether they have some significance in biological systems (Shimshick and McConnell, 1973). A recent review discusses the formation and implications of lipid domain formation in biomimetic as well as biological systems (Kinnun et al., 2020). Of particular relevance for the present study is that sphingomyelin and cholesterol emerge as of particular importance when it comes to domain formation. This motivated our choice to study sphingomyelin, although it has been considered to be only about 5–6% of the brain lipid extract from the gray matter (Dreissig et al., 2009). Domain formation has been demonstrated by using neutron scattering and different isotopic contrasts in the membranes of living *Bacillus subtilis* cells as lateral features of less than 40 nm, considered consistent with the notion of lipid rafts (Nickels et al., 2017).

Neurotransmission has been considered to be facilitated by cholesterol according to several different mechanisms, involving also membrane cluster formation (Milovanovic et al., 2015). These clusters could be imaged thanks to the advancement of super-resolution-stimulated emission depletion (STED) microscopy. Cholesterol levels have also been thought to affect neural diseases like Alzheimer (Fernández-Pérez et al., 2018; Phan et al., 2018; Sparr and Linse, 2019). Increasing cholesterol levels in human embryonic kidney cells and rat hippocampal neurons were found to increase membrane rigidity and promote Aβ-peptide association and aggregation, but reduce membrane disruption (Fernández-Pérez et al., 2018).

Hofmeister or specific ion effects have always been central in biology and affect molecular forces. This has been reviewed in overview by Lo Nostro and Ninham (2012). It is clear that specific ion effects are at play in numerous systems, where they affect the biological function by changes in self-assembled structures. Here, we have also considered the effect of specific ion effects by studying the effect of adding calcium to a physiological sodium chloride solution as well as replacing part of the sodium chloride with lithium chloride. Lithium chloride is administrated as psychopharmacological treatment to reduce the risk of suicide in people with mood disorders (Cipriani et al., 2013). Specific ion effects with lithium salts are enormous, as is illustrated by the behavior of double-chained sulfosuccinates (Karaman et al., 1994). The sodium salts are hydrophobic and form lamellar phase. Lithium salts swell and form spontaneous vesicles. In viscoelastic surfactant-based worm-like micelles, they significantly affect the phase behavior (Tatini et al., 2021).

This study aims to explore the variety of morphologies that brain lipid extracts do form in aqueous dispersions. We have used both extract as well as samples with two important brain lipid constituents, namely, sphingomyelin and cholesterol. We have varied the cholesterol content, salt solution composition, and temperature. The efficiency as salting-out agent for the ions studied follows the order Na^+^ > Li^+^ > Ca^2+^ (Lo Nostro and Ninham, 2012). The objective is to reveal the effect of cholesterol as well as the influence of the ion composition. This study has a bearing on the understanding of the mechanisms of action of bioactive molecules, e.g., pheromones and anesthetics.

## Materials and Methods

The effect of cholesterol on the morphology of aqueous dispersions of brain lipids was investigated. Dispersions of porcine sphingomyelin (56–70 wt%) with varying amounts of cholesterol (0–14 wt%) were prepared in a physiological salt solution (0.9 wt%) (154 mM NaCl and 1 mM CaCl_2_). Also, the effect on salt solution composition was investigated, comparing 0.9 wt% NaCl with a solution at a total ionic strength of 161 mM, comprising 0.8 wt% NaCl (137 mM) and 0.1 wt% LiCl (24 mM). Dispersions of porcine brain lipid extracts were prepared with 70% lipid in 0.9 wt% NaCl and 1 mM CaCl_2_ for SAXD and DSC. In addition, the effect of replacing 11% of the sodium by lithium ions on the aggregate morphology in excess of aqueous solvent was investigated by cryo-TEM. No added buffer ions were used, and the same anion was used, focusing on the effect of the cation. Previous reports have shown an adsorption difference between different anions affecting, e.g., pH measurements (Salis et al., 2006).

The porcine brain lipid sample was extracted from a pig brain obtained from a Tuscany slaughterhouse (Italpork, Ponte Buggianese, Pistoia) and immediately frozen at −24°C. We scratched the cortical, external surface (gray matter). Extraction with 1-butanol/water was conducted using the method described by Pasquini and Soto (1972). The organic solvent was dried over sodium sulfate. The solvent was then evaporated, the samples were first freeze dried, and then kept at -24°C in a freezer. Pasquini and Soto (1972) reported that such an extract contained 38.0% phospholipids, 2.8% galactolipids, 22.6% cholesterol, 5.9% proteolipid proteins, and the remainder not determined. It should be noted that in their study, the phospholipid fraction likely included sphingomyelin and plasmalogen.

The samples were investigated by wide-angle (WAXD) and small-angle X-ray diffraction (SAXD) at different temperatures. Small-angle X-ray diffraction (SAXD) was then used to identify the particular LC structure of the samples. SAXD and WAXD measurements were performed on a Kratky compact system with slit collimation equipped with a position-sensitive detector (OED 50 M, Mbraun, Graz, Austria), containing 1,024 channels, each with a width of 53.6 μm. Cu-Kα radiation of wavelength 1.54 Å was provided by a Seifert ID-300 X-ray generator, operating at 50 kV, and 40 mA. A 10-μm-thick nickel filter was used to remove the Kβ radiation, and a 1.5-mm Wolfram filter was used to protect the detector from the primary beam. The beam width (defined as the beam width where the intensity is half of that at the maximum) was 0.59 mm. The sample-to-detector distance was 277 mm, while the wide-angle detector was placed at an angle of 20.2° and with a sample-to-detector distance of 297 mm. In order to minimize scattering from air and increase signal-to-noise ratio, the volume between the sample and the detector was under vacuum. During the measurements, the samples were placed in a capillary, and the temperature (25°C) was controlled to within 0.1°C by using a Peltier element.

Differential scanning calorimetry (DSC) was performed by means of a DSC-Q2,000 from TA Instruments (Philadelphia, PA, United States). The samples were first cooled from 20 to 60°C at 10°C/min, then heated up to 80°C at 5°C/min, and eventually cooled at the same rate down to -60°C. Measurements were conducted in N_2_ atmosphere, with a flow rate of 50 ml/min.

Dilute dispersions (5–10 wt%) were investigated by cryogenic transmission electron microscopy (cryo-TEM). The temperature (25–28°C) and high relative humidity (close to saturation) in the sample vitrification system was adjusted to avoid evaporation and ensured that proper vitreous film was formed. The dispersions were prepared at 30°C and stored at 40°C until application to the grid. Of the lipid dispersion, 5 μl was placed on the carbon film supported by a cupper grid and blotted with filter paper to reduce film thickness. In the sample vitrification system, the temperature was set to 25–28°C, and a high relative humidity (close to saturation) was maintained. This ensures that evaporation is avoided and that a proper vitreous film is formed. The grid was quenched in liquid ethane at -196°C, stored under liquid nitrogen (-196°C), and transferred to a cryo-TEM (Philips CM120 BioTWIN Cryo) equipped with a post-column energy filter (Gatan GIF 100), using an Oxford CT3, 500 cryo-holder and its workstation. The acceleration voltage was 120 kV, and the working temperature was below −180°C. The images were digitally recorded with a CCD camera (Gatan MSC 791) under low-dose conditions. The under focus was approximately 1 μm.

## Results and Discussion

### Sphingomyelin

#### Dispersions of Sphingomyelin in Physiological Salt Solution With CaCl_2_

**FIGURE 1:**
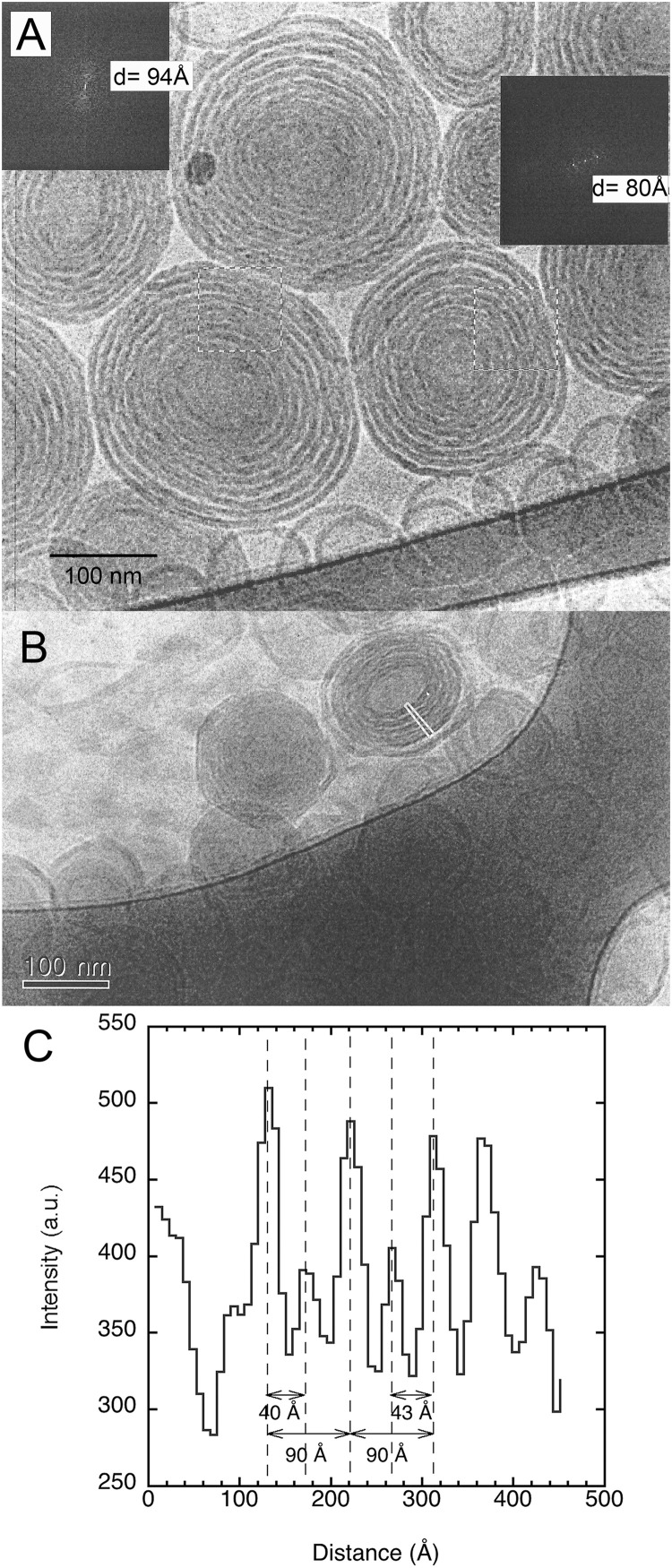
Shows typical cryogenic transmission electron microscopy (cryo-TEM) images of porcine brain sphingomyelin dispersions. The studied dispersion contains 10 wt% lipids in 0.9 wt% (154 mM) NaCl and 1 mM CaCl_2_. **(A)** An image with inserted Fourier transforms of two marked areas that gave repeat distances of 94 and 80 Å, respectively. **(B)** An image of another sample with the same lipid and salt solution composition. The indicated thin rectangle in the image was analyzed in terms of pixel intensity. **(C)** The results of the analysis of the image in the indicated rectangle. The results are shown as a histogram of intensity vs. distance, where high intensity indicates light areas of the image. The image analysis was performed using ImageJ (Schneider et al., 2012).

In the dilute regime, sphingomyelin forms large multilamellar vesicles (Figure 1). Interestingly, these vesicles feature what appear to be double bilayers. Such structures have been reported for biological systems (Eskelinen and Kovács, 2011). They occur too when 15 mol% of the plasmalogen ethanolamine (C16:1p–22:5n6 PE) is incorporated into host dioleoylphosphocholine (DOPC) bilayers (Angelova et al., 2021). Furthermore, the bilayers appear to be rippled, which suggests that this can be a rippled phase rather than a double bilayer. In fact, this has previously been reported for brain sphingomyelin based on freeze-fracture electron microscopy (Meyer et al., 1999). A detailed analysis of the image in Figure 1A, using Fourier transformation of two marked areas, gave a repeat distance of 94 and 80 Å, respectively. Here, we note that this analysis is based on a few bilayers, and as indicated by the results, there is a considerable spread in the data. We here note that this is a projection of a 3D object into a 2D plane, which imposes a degree of uncertainty. The image in Figure 1B is of another sample with the same lipid and salt solution composition where the “double bilayer” feature appears more clearly. The indicated that the thin rectangle in the image was analyzed in terms of pixel intensity, and the resulting histograms are shown in Figure 1C. It should be noted that the high intensity here indicates light areas of the image. For this image, it was possible to resolve areas that were distinctly light, gray, and dark, which can be assigned to different lipid concentrations with the dark area being a high concentration of lipids. The peak-to-peak difference for the light areas is 90 Å, which is close to the value obtained by Fourier transform analysis. The distance from the brightness to the middle of the gray area is about 40–45 Å, depending on the area selected. It is tempting to assume that this might be the extent of the ripples in the ripple phase, but this definitely requires deeper analysis. We note that when these dispersions were prepared at a temperature of about 28°C and equilibrated for 1–2 h at room temperature, the vesicles appear to have a facetted morphology, usually observed for frozen chains.

**FIGURE 2:**
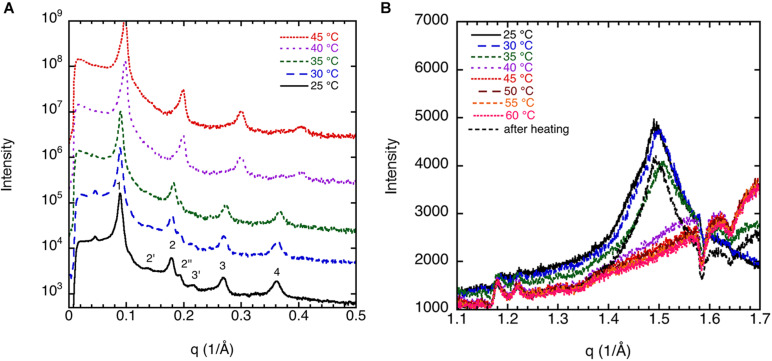
**(A)** Small-angle X-ray diffraction (SAXD) data for 70 wt% porcine brain sphingomyelin in water containing 0.9 wt% (154 mM) NaCl and 1 mM CaCl_2_ recorded at different temperatures. Note that the diffractograms are displaced to facilitate comparison. The indexing of the different peaks (1, 2′, 2, 2″, 3′, 3, and 4) together with their relative intensity are shown in Table 1. **(B)** Wide-angle X-ray diffraction (WAXD) data for 70 wt% porcine brain sphingomyelin in 0.9 wt% (154 mM) NaCl and 1 mM CaCl_2_ recorded at different temperatures upon heating as indicated in the figure. The measurement at 25°C is also repeated after the sample had been heated to 60°C. The peak observed at 25°C and 30°C corresponds to a spacing of 4.20Å, while it decreases slightly to 4.17Å at 35°C. The peak disappears at higher temperature. For the sample remeasured at 25°C after the heating cycle, the value is again 4.20Å.

The corresponding sample at high lipid content (70 w%) gives a diffraction pattern typical for a liquid crystalline lamellar, L_α_, phase or the corresponding gel phase with four well-defined peaks (Figure 2A). The indexing of the different peaks (1, 2′, 2, 2″, 3′, 3, and 4) together with their relative intensity compared with that of peak 1 is shown in Table 1. The additional weak reflections are suggested to indicate the presence of double bilayer or more likely a ripple phase as discussed in conjunction with the cryo-TEM images in Figure 1 above. First, it should be noted that d-spacing decreases with increasing temperature above 35°C, from 70.0 ± 0.5 Å at 25°C to 60.1 ± 0.6 Å at 45°C. The sample heated to 45°C was cooled down again to 25°C, and a new diffractogram was recorded. The lamellar peak appears at the same position, but the intensity of the peaks has decreased (data not shown). This suggests that the swelling behavior with temperature is reversible, but the intensity of the Bragg peaks is not. The decrease in intensity can be attributed to the change in domain size induced by annealing at high temperature. The main reflections 1, 2, 3, and 4 at 25°C give a repeat distance of 70.0 ± 0.5 Å that is significantly smaller than the values extracted from the cryo-TEM data. Again, it should be emphasized that the SAXD was performed on a gel (lamellar) phase sample at low water content (70% lipids), while the cryo-TEM data were recorded at excess salt solution with 10% of lipids. Thus, we expect that the cryo-TEM data would reflect a fully swollen sample and, hence, a larger repeat distance of about 90 Å. When it comes to indexing the satellite peaks 2′, 2″, and 3′, this is more challenging as, in particular, 2′ and 3′ are significantly weaker with a height that is only about 0.1–0.2% of the main peak. If this is the second and third order reflection of a lamellar repeat, this would correspond to a repeat distance of about 90 Å, which is similar to the value observed from image analysis of cryo-TEM data in Figure 1. The additional peak, 2″, corresponds to a repeat distance of about 32 Å. This is about 10 times as intense as the other satellite peaks. In the analysis of the cryo-TEM data, we also observed a repeat distance of 40–45 Å, which corresponds to the position of the 2′, but this might be coincidental. We did not attempt to index the SAXD according to a ripple phase as for the additional reflection peak as discussed by Rappolt and Rapp (1996). It should be noted that the satellite peaks disappear at temperatures above 35°C.

**TABLE 1:** Effect of temperature on the lamellar structures for 70 wt% porcine brain sphingomyelin in water containing 0.9 wt% (154 mM) NaCl and 1 mM CaCl_2_.

**Peak**	**1**		**2′**		**2**		**2″**		**3′**		**3**		**4**		
**T (°C)**	**d (Å)**	**I%**	**d (Å)**	**I%**	**d (Å)**	**I%**	**d (Å)**	**I%**	**d (Å)**	**I%**	**d (Å)**	**I%**	**d (Å)**	**I%**	**a (Å)**
25	70.5	100	*44.8*	*0.2*	35.1	2.5	*32.4*	*2.1*	*28.7*	*0.1*	23.3	0.8	17.4	0.6	70.0 ± 0.5
30	70.5	100	*45.3*	*0.2*	35.1	2.3	*32.6*	*2.8*	*28.7*	*0.1*	23.2	0.6	17.4	0.5	70.0 ± 0.5
35	69.9	100			34.4	2.0	*32.4*	*1.7*			22.9	0.5	17.0	0.3	68.9 ± 0.7
40	64.3	100			31.5	1.8					20.8	0.4	15.4	0.1	62.9 ± 1.1
45	60.8	100			30.1	1.3					20.0	0.7	14.8	0.2	60.1 ± 0.6

*The table shows a summary of the assignments of the reflection peaks from the diffractograms as shown in Figure 2A. Here d = 2π/q is the interlayer spacing for the different reflections 1, 2, 3, 4 corresponding to the lamellar structure. From these values a lamellar repeat distance, a, is calculated and is given ± standard deviation. The peak height I is calculated as percentage of the main reflection peak after subtracting the estimated baseline. Additional peaks are indicated as 2′, 2″, and 3′.*

The peak in the WAXD data (Figure 2B) observed at 25°C and 30°C corresponds to a spacing of 4.20 Å, while it decreases slightly to 4.17 Å at 35°C. The peak disappears at higher temperature. The sharp peak at q ≈ 1.5 Å^–1^ or spacing around ≈4.20 Å in the wide-angle regime is typical for crystalline chains. As the temperature is raised to 40°C, these peaks disappear due to the melting of the chains. It should be noted that the peak reappears if the sample heated to 45°C was cooled down again to 25°C, and a new diffractogram was recorded. The crystallinity of the chains is dependent on cholesterol content but also to some extent on the salt solution composition as will be discussed further below. It is remarkable to notice that the melting temperature (without cholesterol) is around 35–40°C, that is, in the same regime as the physiological temperature (McIntosh et al., 1992).

#### Effect of Cholesterol on Sphingomyelin Dispersions

**FIGURE 3:**
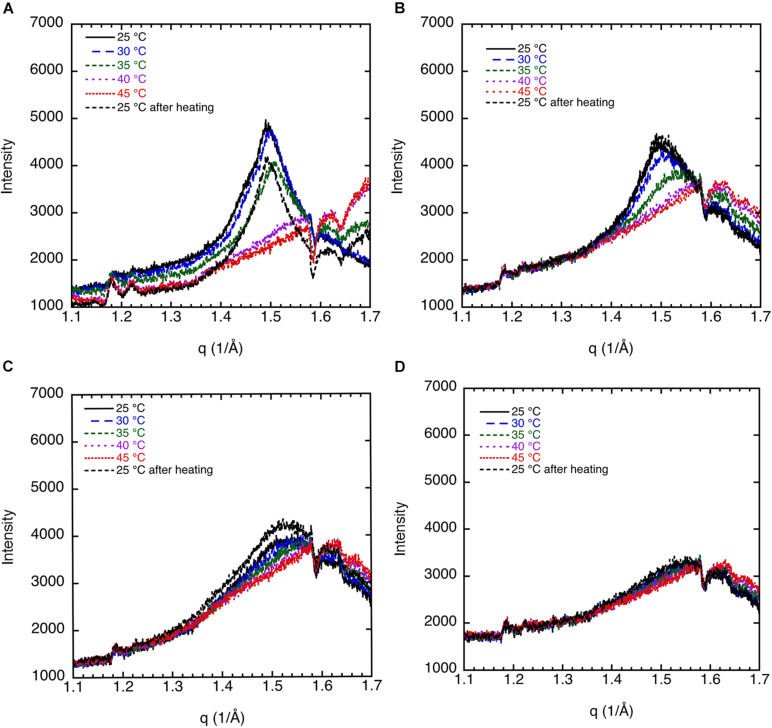
The effect of cholesterol on the crystalline order of porcine brain sphingomyelin in 0.9 wt% (154 mM) NaCl and 1 mM CaCl_2_ recorded at different temperatures by WAXD. **(A)** Sphingomyelin (70%) and no cholesterol (0% of lipid). Peak position corresponds to spacing of 4.20 Å at 25°C and 30°C, 4.17 Å at 35°C, while no peak at higher temperatures. **(B)** Sphingomyelin (65.1%) and 4.9% cholesterol (7% of lipid). Peak position corresponds to spacing of 4.20 Å at 25°C, 4.17 Å at 30°C, rounded peak at 35°C, while no peak was observed at higher temperatures. **(C)** Sphingomyelin (59.5%) and 10.5% cholesterol (15% of lipid). A rounded peak appears at 25°C, while no peak was observed at higher temperatures. **(D)** Sphingomyelin (56%) and 14% cholesterol (20% of lipid). No defined peak appears at any temperature.

The effect of cholesterol on the crystalline order of porcine brain sphingomyelin in 0.9 wt% (154 mM) NaCl and 1 mM CaCl_2_ is recorded at different temperatures by WAXD, and the results are presented in Figure 3. Both without cholesterol and when 7% of the lipids consist of cholesterol, the chain melting appears at around 35–40°C as is apparent from the diffractogram. Cholesterol is expected to introduce disorder in the sphingomyelin gel phase, which is manifested in the less sharp WAX peak at q ≈ 1.5 Å^–1^ at higher cholesterol contents. When 15% of the lipid is cholesterol, a rounded peak appears at 25°C, while no peak occurs at higher temperatures (Figure 3C). At a cholesterol content of 20%, there is essentially no clear identifiable peak in the WAXD scattering region, indicating that the chain-melting transition occurs below 25°C (Figure 3D). It is clear then that the WAXD diffractogram recorded at different temperatures and cholesterol content reflect the effect of cholesterol on the chain-melting transition.

**FIGURE 4:**
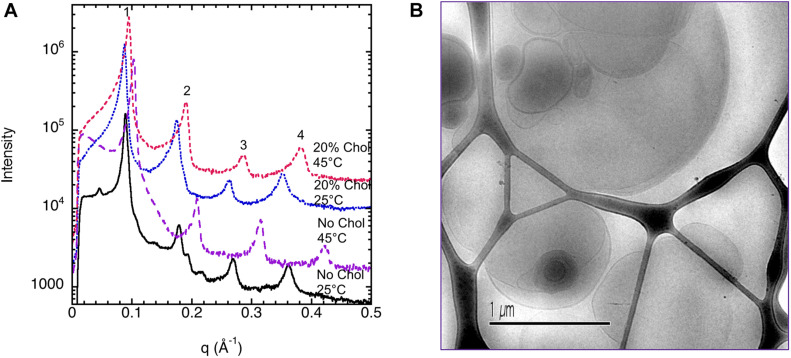
Shows the effect of cholesterol on the ordering of porcine brain sphingomyelin in 0.9 wt% (154 mM) NaCl and 1 mM CaCl_2_ recorded at 25°C and 45°C. **(A)** SAXD data for 70% sphingomyelin and no cholesterol (0% of lipid) as well as 56% sphingomyelin and 14% cholesterol (20% of lipid) are shown. The interlayer spacing for the different reflections and the relative intensities of the Bragg peaks are summarized in Table 2. Note that the intensities of the diffractograms are scaled for better clarity. **(B)** A typical cryo-TEM image of porcine brain sphingomyelin dispersions in the presence of cholesterol. As opposed to in the absence of cholesterol (Figure 1), the image features unilamellar vesicles of different sizes. The studied dispersion contains 10 wt% lipid in 0.9 wt% (154 mM) NaCl and 1 mM CaCl_2_, where 7 wt% of lipids are cholesterol.

The SAXD data for the sphingomyelin/cholesterol system that illustrates the effect of cholesterol is shown in Figure 4A. Table 2 shows a summary of the assignments of the reflection peaks from the diffractograms together with the relative peak intensity. Here, we have chosen to show data for samples with 30% of salt solution (154 mM NaCl and 1 mM CaCl_2_) without cholesterol and when 20% of the lipids consist of cholesterol as we obtain the most resolved diffractogram and largest effect of cholesterol under these conditions. At 25°, we observe a repeat distance of 70.1 ± 0.5 Å without cholesterol. Above the chain-melting temperature, about 40°C as observed in the WAX diffractograms (Figure 3), the satellite peaks disappear as is apparent from the data recorded at 45°C (Figure 4A) and discussed above. It is also noteworthy that the d-spacing decrease, from 70.0 ± 0.5 Å, with increasing temperature to 60.1 ± 0.6 Å. This amounts to a decrease of nearly 10 Å, which might be assigned to the larger mobility of the chains due to partial melting and thinner bilayer or dehydration of the lamellar phase. The addition of 20% cholesterol removes the double peaks observed in the diffractogram for sphingomyelin without cholesterol already at low temperature. Figure 4A and Table 2 show that the d-spacing is slightly larger for the system with 20% of cholesterol, i.e., 71.7 ± 0.1 Å compared with 70.0 ± 0.5 Å without cholesterol. We also note that the effect of temperature on d-spacing is less pronounced with a decrease in the value of about 6 Å to 65.9 ± 0.4 Å. Another interesting observation is that the height, i.e., intensity of the peak corresponding to the second order reflection increases about five times. This is consistent with an increased order of the phase induced by cholesterol as frequently reported for phospholipid systems (Hjort Ipsen et al., 1987; Mortensen et al., 1988).

**TABLE 2:** Effect of adding cholesterol (20 wt% of lipid) on the lamellar structures of 70 wt% porcine brain sphingomyelin in water containing 0.9 wt% (154 mM) NaCl and 1 mM CaCl_2_.

**Peak**	**1**		**2**		**3**		**4**		
**Conditions**	**d (Å)**	**I%**	**d (Å)**	**I%**	**d (Å)**	**I%**	**d (Å)**	**I%**	**a (Å)**
25°C	70.5	100	35.1	2.5	23.3	0.8	17.4	0.6	70.0 ± 0.5
25°C, 20% Cholesterol	71.8	100	35.9	9.4	23.9	0.8	17.9	1.3	71.7 ± 0.1
45	60.8	100	30.1	1.3	20.0	0.7	14.8	0.2	60.1 ± 0.6
45°C, 20% Cholesterol	66.4	100	33.0	7.0	21.9	0.8	16.4	1.3	65.9 ± 0.4

*The table shows a summary of the assignments of the reflection peaks from the diffractograms shown in Figure 4A. Here d = 2π/q is the interlayer spacing for the different reflections 1, 2, 3, and 4 corresponding to the lamellar structure. From these values, a lamellar repeat distance, a, is calculated and is given as ± standard deviation. The peak height I is calculated as percentage of the main reflection peak after subtracting the estimated baseline. Values for the additional peaks 2^′^, 2″, and 3^′^ in the sample without cholesterol at 25°C (absent in other samples) are shown only in Table 1.*

We also note that, already with 7% of the lipids exchanged for cholesterol, the morphology of the dispersed aggregates changes profoundly as is observed by comparing Figure 1, 4B. The electron micrographs show that with cholesterol, the apparent double bilayer multilamellar vesicles are replaced with large vesicular type of structures with smooth bilayers and of different sizes.

#### Effect of Salt Solutions on Sphingomyelin

**FIGURE 5:**
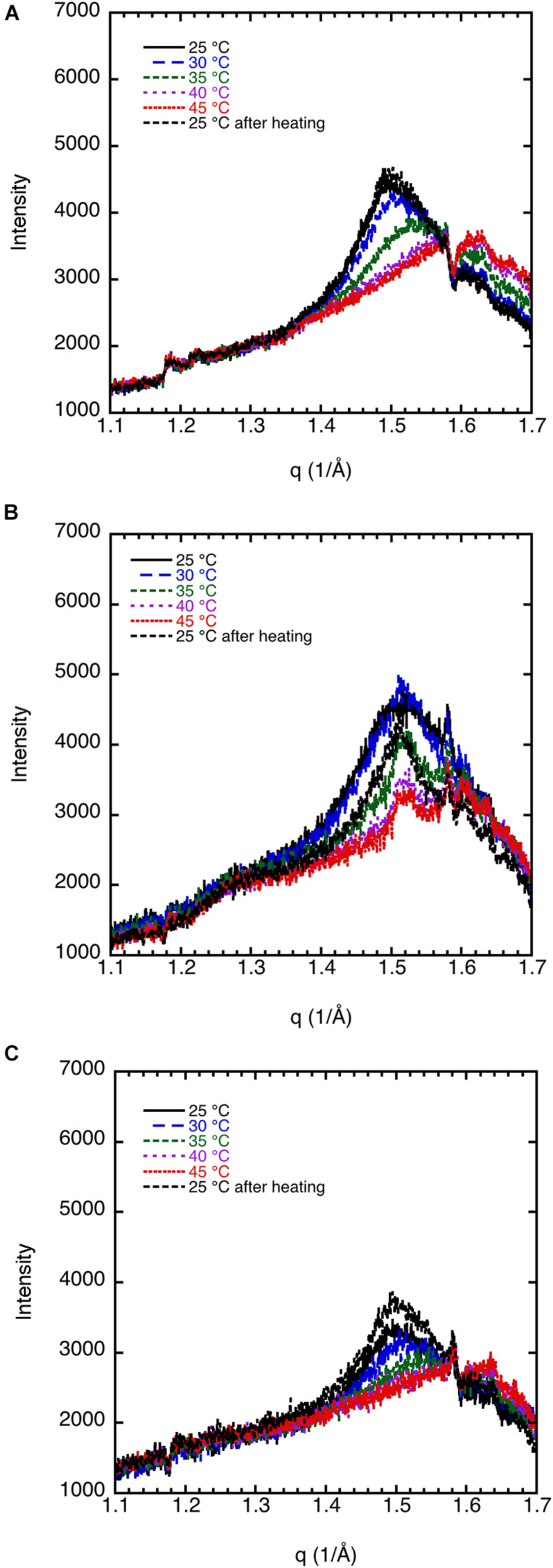
WAXD data show the effect of salt composition on the ordering of porcine brain sphingomyelin, 65.1% sphingomyelin, and 4.9% cholesterol (7% of lipid) recorded at different temperatures. **(A)** In 0.9 wt% (154 mM) NaCl and 1 mM CaCl_2_, the peak position corresponds to spacing of 4.20 Å at 25°C, 4.17 Å at 30°C, rounded peak at 35°C, while no peak at higher temperatures. **(B)** In 0.9 wt% (154 mM) NaCl, the peak position corresponds to a spacing of 4.15 Å at 25–35°C, and no clear peaks at higher temperature. **(C)** In 0.8 wt% (137 mM) NaCl and in 0.1 wt% (24 mM) LiCl, the peak position corresponds to a spacing of 4.20 Å at 25°C, a less defined peak at 4.15 Å at 30°C, and no peaks at higher temperatures. Note that the intensity is scaled so to obtain the same background for all diffractograms.

We discuss the effect of the type of salt on the chain melting transition as observed by WAXD. Key data are shown in Figure 5. The 70% sphingomyelin sample with 1 mM CaCl_2_ in the 0.9 wt% (154 mM) NaCl salt solution gives a sharper 4.20 Å peak than for the other conditions. This might be due to the fact that divalent cations are known to bind strongly with, e.g., phospholipids (McLoughlin et al., 2005). We note also that the chain-melting transitions seem to occur at a slightly higher temperature with CaCl_2_ (≈40°C) than with only monovalent salt. The WAXD peak becomes less defined when part of the NaCl is replaced with LiCl with a peak intensity decrease of about 75%. The chain-melting transition also seems to occur at a lower temperature of 30–35°C in the presence of LiCl, compared with 35–40°C in neat NaCl of the same ionic strength. Here, we again note that Na^+^ is more efficient as a salting-out ion than Li^+^ (Lo Nostro and Ninham, 2012), which might explain the slightly higher chain-melting temperature with only NaCl. This is presumably due to the different hydration-free energy of the two cations: for Li^+^ and Na^+^ Δ*_*hydr*_G* is -475 and -365 kJ/mol, respectively (Marcus, 1991).

**FIGURE 6:**
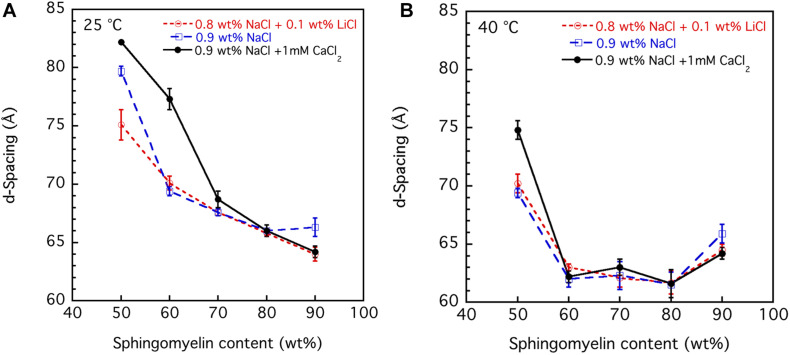
The effect of salt solution composition, i.e., 0.9 wt% (154 mM) NaCl and 1 mM CaCl_2_, 0.8 wt% (137 mM) NaCl and 0.1 wt% (24 mM) LiCl, and 0.9 wt% (154 mM) NaCl, on the interlamellar spacing (d-spacing) obtained from SAXD data as a function of porcine brain sphingomyelin content recorded at two different temperatures, **(A)** 25°C and **(B)** 40°C. The obtained values are the mean values calculated from all observed first to fourth order of reflections. For the samples with the largest hydration (50 wt% lipid), the third and fourth order peaks are very broad, and therefore, the q value of the peak is uncertain. In those cases, we used only the first and second order reflections. The error bars in the graph are the standard deviation of the calculated mean values.

The SAXD data obtained by studying the swelling in the presence of different salt solution conditions are summarized in Figure 6. The values obtained are the mean values calculated from all observed first to fourth order of reflections, and the error bar is the standard deviation. It should be noted for the samples with the largest hydration (50 wt% lipid) that the third and fourth order peaks are very broad. This suggests that under these conditions, the system is close to the swelling limit. The swelling appears to be non-ideal, i.e., it does not follow the one-dimensional swelling law for a lamellar phase (Luzzati et al., 1960). This is particularly apparent at 40°C, which will be discussed further in the next section. We note that the d-spacing values tend to be lower in the presence of the NaCl + LiCl solution. The largest effect is observed at the lower sphingomyelin content. This is consistent with the lowering of the chain-melting temperature in the presence of Li^+^ ions (Figure 5), which implies slightly more disordered acyl chains of the lipids. We also note that in the presence of the Ca^2+^, which raises the chain-melting temperature slightly, the largest d-spacing is observed. This is consistent with more stretched out lipid chains. When the chains are in the fluid state less (Figure 6B), almost no differences between the salts are observed. Also, the value for the d-spacings is different for the different salt solutions.

#### Swelling Behavior of Sphingomyelin

**FIGURE 7:**
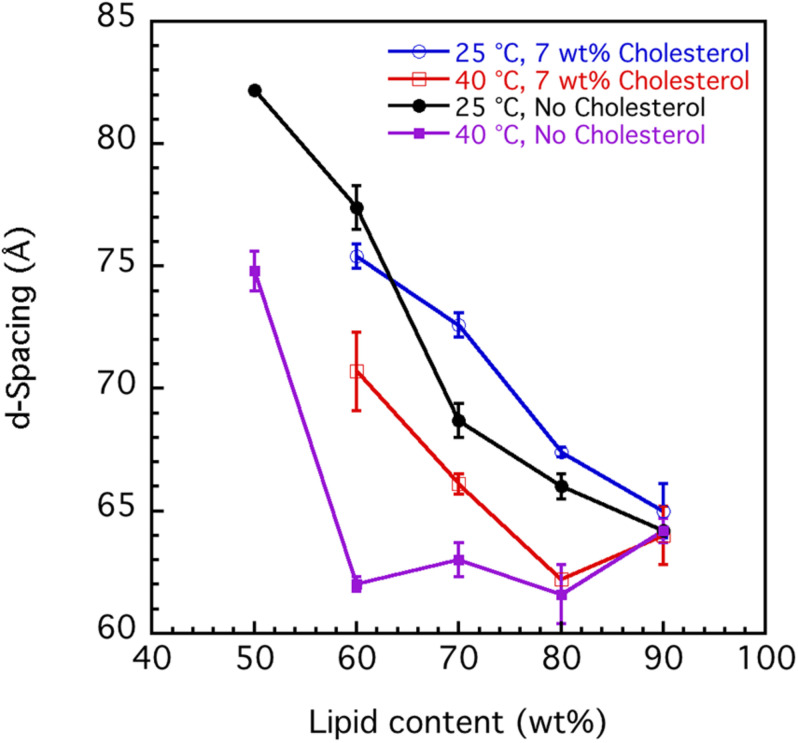
Effect of cholesterol on the interlamellar spacing (d-spacing) obtained from SAXD data as function of porcine brain sphingomyelin content recorded at two different temperatures, 25°C and 40°C. The swelling curves for sphingomyelin in 0.9 wt% (154 mM) NaCl and 1 mM CaCl_2_ without cholesterol and where 7 wt% of lipid fraction was cholesterol are shown. The obtained values are the mean values calculated from all observed first to fourth order of reflections. For the samples with the largest hydration (50 wt% lipid), the third and fourth order peaks are very broad, and the q value of the peak is, therefore, uncertain. In those cases, we used only the first and second order reflections. The error bars in the graph are the standard deviations of the calculated mean values.

The swelling curves in Figure 6 suggest non-ideal swelling behavior within the concentration range investigated. This is particularly evident at 40°C (Figure 6B), where the d-spacing does not change with lipid content above 60 wt%. It is possible to make a good linear fit of d-spacing vs. the inverse volume fraction of lipid for the data recorded at 25°C, but the slopes give unreasonable bilayer thickness of 15–20 Å (Luzzati et al., 1960). In Figure 7, we present data that illustrate the effect of cholesterol on the swelling behavior in 0.9 wt% (154 mM) NaCl and 1 mM CaCl_2_. Remarkably, cholesterol has very little effect on the swelling behavior at 25°C. At 40°C, the d-spacing seems to increase with the addition of cholesterol. This is presumably because of the slightly different locations of the cholesterol at the two temperatures. At the higher temperature, the more fluid chains allow penetration of the linear cholesterol molecule deeper into the lipid hydrocarbon chains, so changing effective hydrocarbon volume and increasing head group hydration.

McIntosh et al. studied the thermal, structural, and cohesive properties of bovine brain sphingomyelin (BSM) and N-tetracosanoylsphingomyelin (C24-SM) with and without cholesterol (McIntosh et al., 1992). Their results are rather consistent with our findings, although they only studied the neat, hydrated sphingomyelin and with a 0.5-mol fraction of cholesterol. The acyl chains of C24-SM were found to be tilted and partially interdigitated, which means that the long C24 chain in one bilayer leaflet faces the shorter (unsaturated) sphingosine chain in the other reversed leaflet. This conclusion was made based on the distance between the head groups of about 51 Å in the diffraction pattern, while the distance should be about 57 Å based on the molecular structure. The possible formation of a ripple phase as found for phosphatidylcholine was also discussed but could not be verified with the data presented. Our cryo-TEM images with rippled structure associated with what appears as a double bilayer structure seems to indicate such a rippled structure. This is also consistent with a previous report for brain sphingomyelin based on freeze fracture electron microscopy (Meyer et al., 1999). McIntosh et al. also noted that introducing 50% cholesterol seems to remove the tilt and make the acyl chains disordered. Similar to our findings, they found that the wide-angle X-ray diffraction reflection at 4.15 Å disappears at high cholesterol content, also observed in our present study.

### Brain Extract

#### Dispersion of Brain Extract at High Dilutions

**FIGURE 8:**
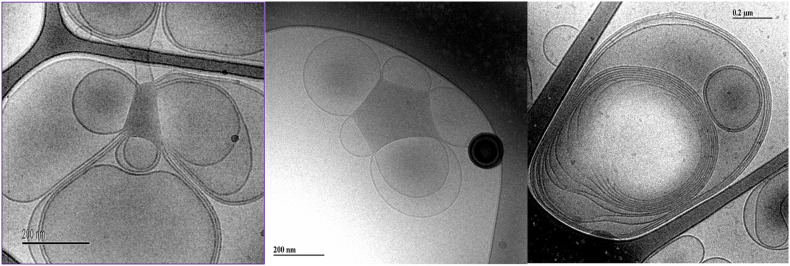
Typical cryo-TEM images of a 10 wt% brain lipid extract dispersion in 0.9 wt% (154 mM) NaCl and 1 mM CaCl_2_. The images show large multilamellar vesicles often attached to a body of higher electron density.

The images of dilute dispersions of brain lipid extract do not feature double bilayer-type vesicles. It rather form large multilamellar vesicles often attached to a body of higher electron density (Figure 8). Similar patterns are not uncommon for dispersed lipid systems containing mixtures of lamellar forming and inverse phase forming lipids (Wadsäter et al., 2018). Recently, a study shows that self-assembled nanoparticles mainly composed of docosapentaenoyl (DPA)-diacyl phosphoinositol (22:5n6–22:5n6 PI)/monoolein (MO)/vitamin E formed multicompartment and multiphase structures similar to the ones shown in Figure 8 (Angelova et al., 2021). They suggested that these are built up by dense regions, most likely with lipid mixtures rich in PUFA-phospholipid and vesicular structures. This was suggested to be a consequence of the de-mixing of the different types of lipids. Here we note that apart from lamellar-forming phosphatidylcholine (about 13%), this type of extract also contains about 24% of reverse phase-forming phosphatidylethanolamine (Dreissig et al., 2009). Furthermore, about 20% of the brain phospholipids are plasmalogens, a unique class of phospholipids that contain a fatty alcohol with a vinylether bond at the sn-1 position and enriched in polyunsaturated fatty acids (Braverman and Moser, 2012). In addition, the extracted brain lipids have a high content of cholesterol, so that they are expected to be in the liquid crystalline state. This is verified by the smooth bilayer structures in the Cryo-TEM images (Figure 8).

#### Liquid Crystalline Phases of Brain Lipid Extracts

**FIGURE 9:**
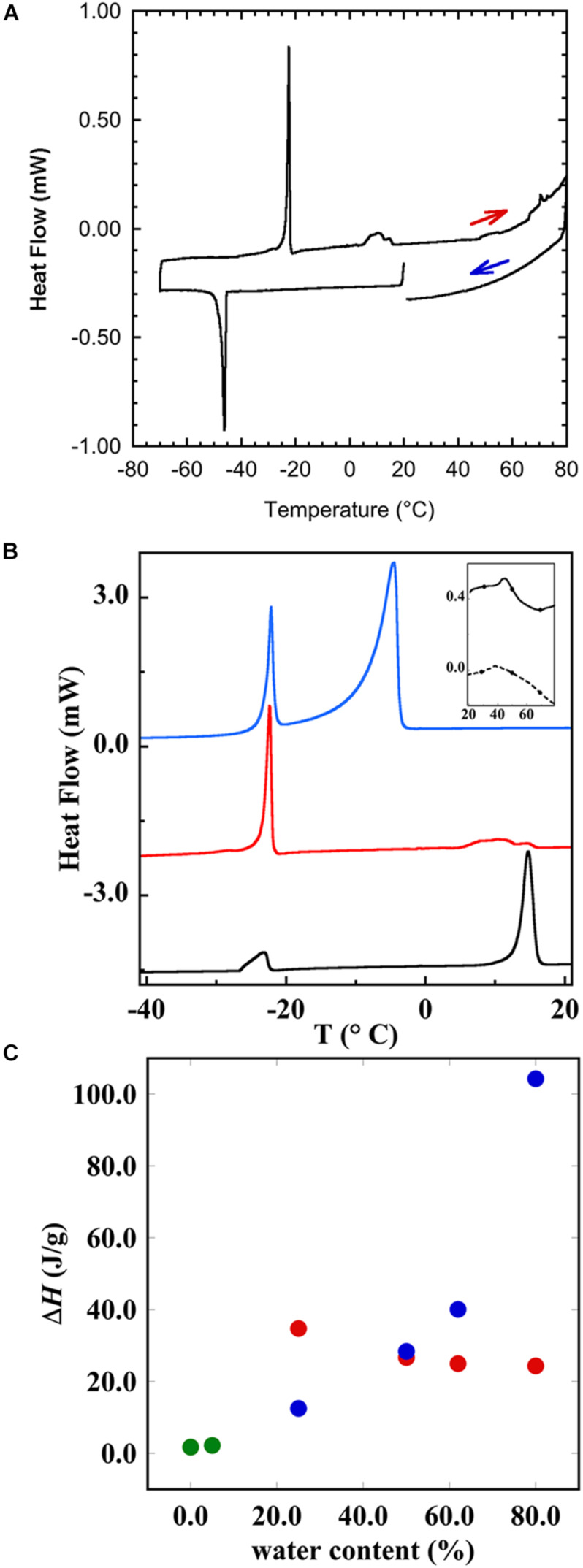
Differential scanning calorimetry (DSC) showing the thermal response of brain lipid extract in 0.9 wt% NaCl and 1 mM CaCl_2_. **(A)** The thermogram of 50 wt% brain lipid extract in 0.9 wt% NaCl and 1 mM CaCl_2_, where the heating and cooling cycles are indicated. The vertical line indicates the start of the cooling—heating—cooling cycle. The samples were first cooled from 20°C to -60°C at 10°C/min, and then heated up to 80°C at 5°C/min and eventually cooled at the same rate down to 20°C. **(B)** The thermograms upon heating, where the different curves correspond to different aqueous content [salt solution with 0.9 wt% (154 mM) NaCl and 1 mM CaCl_2_] of 25 wt% (black), 50 wt% (red), and 80 wt% (blue)]. The inset shows the thermograms obtained from the pure brain lipid extract (full line) and from its hydrated phase (5 wt% of salt solution). **(C)** The enthalpy of the different transition peaks from **(B)** as a function of salt solution content, where green circles refer to the data for pure lipid, and 5 wt% showing only one peak. The other samples show two peaks, one at very low temperature (≈-22°C), and the corresponding enthalpy change is shown as red circles, and the high temperature transition peak is shown as blue circles. The results with onset and peak temperatures and the corresponding enthalpies are summarized in Table 4.

**FIGURE 10:**
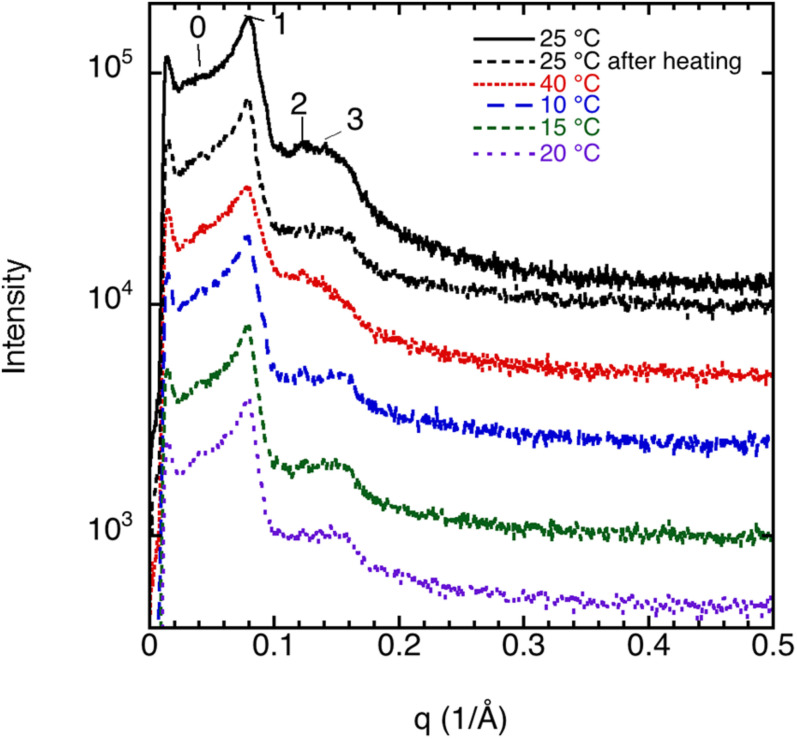
SAXD data for 70 wt% brain lipid extract in 0.9 wt% NaCl and 1 mM CaCl_2_ as function of temperature. The interlayer spacing of the different peaks (0, 1, 2, and 3), together with their relative intensity, is shown in Table 3. Here peak 1 is the only well resolved peak with the highest intensity, while the 0 peak appears as a shoulder. Note the different diffractograms are displaced to facilitate comparison.

The liquid crystalline state is doubly confirmed by the DSC measurements, where a typical thermogram is presented in Figure 9A. The vertical line indicates the start of the cooling—heating—cooling cycle. The samples were first cooled from 20 to −60°C at 10°C/min, and then heated up to 80°C at 5°C/min and eventually cooled at the same rate down to 20°C. We note here that there is a chain-melting peak at about -20°C. There are some smaller peaks around 10°C in the thermogram suggesting some sort of phase transition. To clarify the nature of this transition, SAXD was used to investigate the brain lipid samples further. The results for 70 wt% brain lipid extract in 0.9 wt% (154 mM) NaCl and 1 mM CaCl_2_ as a function of temperature are shown in Figure 10. Table 3 shows a summary of the assignments of the reflection peaks from the diffractograms. Four peaks were identified, and the peak with the highest intensity is labeled as 1 and is the only peak that can be assigned a d value with high accuracy. We note that the diffraction pattern is rather complex verifying the multiphase structure that is apparent from the cryo-TEM images in Figure 8. The SAXD pattern features several peaks, which are preserved during the temperature cycles. Some can probably be assigned to lamellar-like structures. The pattern features a main peak (1) around 80 Å as well as one peak (0) at about 145 Å and another (3) at 44.5 Å not perfectly matching the first three peaks of a lamellar phase. If we compare with the cryo-TEM image, we note that the repeat distance is slightly more than 100 Å compared with 80 Å extracted from the main peak (1) at 70 wt% lipid. The discrepancy compared with the main peak is likely due to the fact that cryo-TEM images were captured on 10-wt% lipid sample, but SAXD data was recorded using a lipid concentration of 70 wt%. Hence, the lamellar phase is expected to be more swollen in the cryo-TEM sample. We also note that the peaks at 50.1 and 44.5 Å shifted to lower spacing when the sample is heated to 40°C. Cooling down to 10°C gives only small changes to the peak positions. It should be noted that a sample that has been heated to 45°C shows the same diffraction pattern when re-run at 25°C as when the sample was first investigated at 25°C. It is clear that further structural analysis is needed to reveal the details of this very intriguing structure.

**TABLE 3:** SAXD data for 70 wt% brain lipid extract in 0.9 wt% (154 mM) NaCl and 1 mM CaCl_2_ as a function of temperature.

***Peak***	***0***		***1***		***2***		***3***	
**Conditions**	**d (Å)**	**I%**	**d (Å)**	**I%**	**d (Å)**	**I%**	**d (Å)**	**I%**
25°C	145	28	79.7	100	50.1	17	44.5	16
25°C, after heating to 40°C	144	37	79.7	100	50.5	28	43.1	13
40°C	145	37	78.9	100	55.4	28	48.5	35
10°C	145	38	79.7	100	52.1	13	41.1	12
15°C	142	34	80.5	100	49.5	13	40.6	12
20°C	140	38	79.7	100	51.4	14	43.1	13

*The table shows a summary of the assignments of the reflection peaks from the diffractograms shown in Figure 10. Here d = 2π/q is the interlayer spacing for the different reflections. Four peaks were identified, and the peak with highest intensity is labeled as 1 and is the only peak that can be assigned a d value with high accuracy. The peak height I is calculated as percentage of the main reflection peak after subtracting the estimated baseline.*

**TABLE 4:** The differential scanning calorimetry (DSC) data for brain lipid extract in 0.9 wt% (154 mM) NaCl and 1 mM CaCl_2_ as function of aqueous content.

**Transition**	**T_*peak*_ (°C)**		**T_*o*__*nset*_ (°C)**		**Δ H (J/g)**	
**Water content (wt%)**	**Low T**	**High T**	**Low T**	**High T**	**Low T**	**High T**
0	–	41.2	–	23.8	–	1.74
5	–	46.5	–	26.5	–	2.22
25	-22.0	10.6	-39.5	3.3	34.8	12.5
50	-22.3	10.6	-23.3	5.0	26.7	9.7
62	-21.6	-9.4	-22.8	-15.0	24.9	40.1
80	-22.1	-4.5	-23.0	-8.6	24.4	104.3

*The results with peak (T_*peak*_) and onset (T_*o*__*nset*_) temperatures as well as the corresponding enthalpies, ΔH of the transition are shown and determined from the thermogram in Figure 9. The baseline was corrected to calculate the onset and peak temperature of the transition as well as the enthalpy.*

#### Effect of Salt Solution on Brain Lipid Dispersions

**FIGURE 11:**
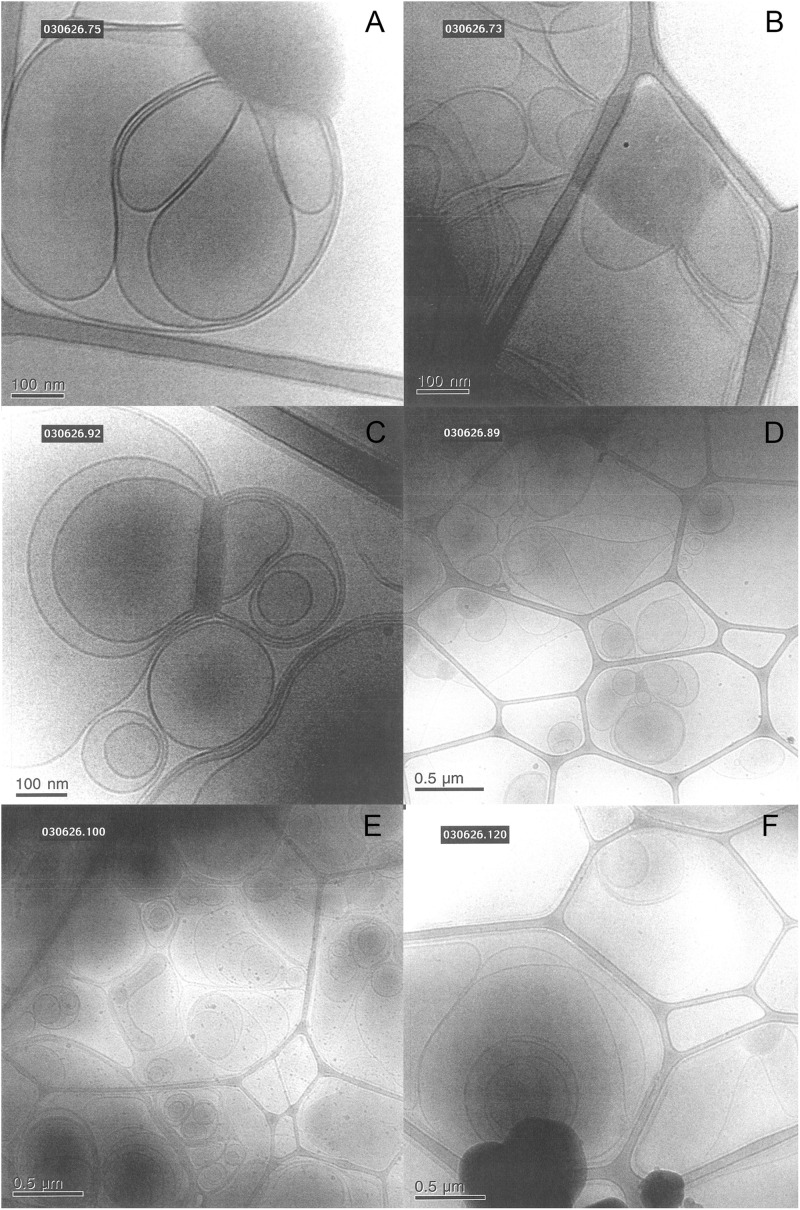
Shows typical cryo-TEM images of 5 wt% brain lipid extract dispersion in aqueous solution with different types of ions, but roughly the same ionic strength. **(A,B)** The images from two different grids of a sample dispersed in 0.9 wt% (154 mM) NaCl and 1 mM CaCl_2_ salt solution, featuring lamellar structures connected to more dense regions not fully resolved. **(C,D)** The images from two different grids from a sample dispersed in 0.9 wt% NaCl (154 mM) salt solution, featuring lamellar structures connected to more dense regions not fully resolved. **(E,F)** The images from two different grids from a sample dispersed in a salt solution containing 0.8 wt% (137 mM) NaCl and 0.1 wt% (24 mM) LiCl, featuring only lamellar structures.

We also investigate the effect of different salt solutions on the morphology of the structure formed in dispersions of porcine brain lipids. In this case, the 5 wt% lipid dispersions were prepared using 0.9 wt% NaCl and 1 mM CaCl_2_, 0.9 wt% NaCl, 0.8 wt% NaCl with 0.1 wt% LiCl similar to the sphingomyelin fraction. Typical images are shown in Figure 11, and we note that there are significant effects of salt composition on the morphologies of the aggregates formed. Similarly, as for the sphingomyelin sample, the largest effect was seen by replacing some of the NaCl in the solution by LiCl. Here, we observed only a vesicular type of structure, contrary to the samples with NaCl and NaCl with added CaCl_2_, where denser aggregates connected to the vesicular structures were formed. The number of these denser aggregates was somewhat higher in the presence of CaCl_2_. This is quite remarkable as it implies that the dense region observed under other ion compositions are not formed in the presence of LiCl under the conditions employed. It is tempting to suggest that the LiCl has a particularly strong effect on the PUFA type of lipids as they are the ones thought to be responsible for the dense regime of the sample as reported by Angelova et al. (2021).

#### Effect of Hydration on Brain Lipid Dispersions

The brain extract samples were dispersed in water at four different concentrations, and their thermal behavior was investigated through DSC and the results are shown in Figures 9B,C and summarized in Table 4. Figure 9B shows the thermogram of three aqueous dispersions of the brain extract, while the inset shows the DSC runs for the pure lipid sample and its hydrated sample (5 wt% of water). In the whole range of temperature investigated, the pure brain lipid (see the black solid line in the inset in Figure 9B) shows an endothermic phase transition at about 44.7°C, probably due to the melting of the sample. The hydrated sample (dotted line in the inset) shows a similar behavior with a significant broadening of the signal, centered at 38°C. When the amount of water increases from 25 to 80 wt%, the higher temperature peak progressively shifts to lower temperature and disappears: it is centered at 14.6°C in the 25 wt% sample and is almost vanished at 50 wt% (with some small contributions around 10°C). Interestingly, in the 25 wt% sample (black curve), there seems to be no free water in the sample, but only interstitial freezable bound water that melts at about -23°C. By increasing the water content up to 50 wt% (red curve), the amount of freezable bound water increases significantly, and a melting temperature remains at –22°C. At 80 wt%, we recorded also the presence of freezable water that melts at −4.7°C.

Figure 9C shows the enthalpy as a function of brain lipid content. In the pure lipid and in the samples with 5 wt% salt solution, the peak temperature (Table 4) and the corresponding enthalpy change are practically the same (green circles). Starting from the sample with 25 wt% salt solution, interestingly, we see two different peaks in each thermogram. One occurs at very low temperature, around -22°C, and another at higher temperature. For the former, the temperature at which the transition occurs does not change with the lipid concentration, while the enthalpy change slightly decreases as the amount of water increases from 25 to 80% (red circles). The other transition instead is accompanied by a much higher enthalpy change that remarkably increases when the water amount is higher than 50% (blue circles).

These results seem to indicate that hydration significantly modifies the thermal behavior of the brain lipids, in accordance with the structural and morphological investigations. Apparently, at least up to a water content of 50 wt%, water molecules are part of the nanostructures built up by bilayers and strongly bound (as non-freezable bound and interstitial water) to the polar head groups. Here, we would like to note that Gershfeld and coworkers, in a number of studies, have shown that the thermal response of the brain lipid, which in turn depends on the composition, can have physiological implications (Ginsberg et al., 1993). Their studies indicate that when the disease affects membrane lipid composition, the membrane can be destabilized at physiological temperatures.

## Concluding Remarks

At first sight, the observations we have made are most peculiar. However, they are predicted by the modern theory of lipid self-assembly (Ninham et al., 2017a). It is a matter of simple geometry that any closed lipid membrane bilayer, spherical, or cylindrical faces a packing problem. The curved bilayer is necessarily unsymmetric. The outer monolayer has positive curvature; the inner layer necessarily has negative curvature. The bilayer is asymmetric. Correspondingly, the exterior and interior physicochemical conditions are also asymmetric. For a multilayered structure, there comes a point when the inner bilayer can no longer pack. This occurs at a radius of about 200 nm for typical biological lipids. The interior lipid bilayers can adjust to this tension by forming a cubic phase (Larsson, 1989; Ninham et al., 2017b).

States of supra-self-assembly become the rule rather than the exception in biological systems. With mixed lipids, phase separation within a bilayer takes place for the same reasons, with mesh phases—2D cubic phases with catenoidal holes and rafts automatically a consequence of packing constraints (Hyde et al., 1997). Transitions between one state of self-assembly to another, as for transmission of the nervous impulse, occur with ease by the Bonnet transformation (Larsson, 1989; Hyde et al., 1997; Ninham et al., 2017b).

The self-assembled structures demonstrated in this study exhibit such states of supra-self-assembly explicitly. It is clear that further analysis is needed to reveal the details of these very intriguing structures. This can most easily be achieved by changing head group hydration interactions using Hofmeister effects and with different salt solutions. The different morphologies seen under different external solution conditions begin to provide insights into phase changes of brain lipids that are coupled to, and are the source and sink of channel formation for ion exchange of sodium, potassium, and other ions that accompany and drive transmission of the nervous impulse. The effect of salt composition in the solution reflected in changes in lipid assembly morphology is due to specific competitive ion adsorption only, physical not biochemistry. This points at the possible connection to the physiological mechanism behind the successful use of lithium salts for the treatment of bipolar disorder.

## Data Availability Statement

The raw data supporting the conclusions of this article will be made available by the authors, without undue reservation.

## Author Contributions

TN drafted the manuscript, participated in the sample preparation, and X-ray diffraction analysis. VA participated in writing the manuscript, performed sample preparation and X-ray diffraction analysis as well as performed all cryo-TEM imaging. PL initiated the study, prepared the brain lipid, performed the DSC measurements and analysis, and participated in writing the manuscript. BN inspired the study, participated in the analysis of the data, and writing of the manuscript. All authors contributed to the article and approved the submitted version.

## Conflict of Interest

The authors declare that the research was conducted in the absence of any commercial or financial relationships that could be construed as a potential conflict of interest.

## Dedication

This article is dedicated to the Memory of Prof. Kåre Larsson, 1937–2018, who inspired this study.
